# Impact of Screw Insertion Technique on Thoracic Pedicle Screw Anchorage: Biomechanical Comparison of the Modified Slide, Slide, Funnel, and Conventional Techniques

**DOI:** 10.1002/jsp2.70202

**Published:** 2026-06-23

**Authors:** Richard A. Lindtner, Fabian Krumm, Werner Schmoelz, Julian Benko, Andreas E. Ellmerer, Johannes Dominikus Pallua, Romed Hoermann, Anna Spicher

**Affiliations:** ^1^ Department of Orthopaedics and Traumatology Medical University of Innsbruck Innsbruck Austria; ^2^ Division of Clinical and Functional Anatomy, Department of Anatomy, Histology and Embryology Medical University of Innsbruck Innsbruck Austria

**Keywords:** biomechanics, cadaveric study, cyclic loading, funnel technique, modified slide technique, pedicle screw anchorage, pedicle screw insertion technique, pedicle screws, spinal instrumentation, thoracic vertebrae

## Abstract

**Background:**

Several techniques have been developed to facilitate safe and accurate thoracic pedicle screw placement. However, their impact on screw anchorage has not been systematically evaluated. This study compared thoracic pedicle screw anchorage following placement using the Modified Slide, Slide, Funnel, and Conventional techniques under cyclic loading.

**Study Design:**

Cadaveric biomechanical study.

**Methods:**

Forty‐five fresh‐frozen human thoracic vertebrae (T4–T11) were allocated to three experimental groups (*n* = 15 each). For standardized paired comparisons, one randomly selected pedicle of each vertebra was instrumented using the Modified Slide technique, developed by the authors as a refinement of the original Slide technique, and the contralateral pedicle with one of three established techniques. Screws were subjected to cyclic craniocaudal loading until loosening or a maximum load level of 750 N.

**Results:**

Screws placed using the Modified Slide technique withstood 1.9‐fold and 1.5‐fold more load cycles until loosening than those placed with the Slide and Funnel techniques, respectively (both *p* < 0.001), but only 0.9‐fold that of screws inserted with the Conventional technique (*p* = 0.008).

**Conclusions:**

The Modified Slide technique preserves the advantage of the Funnel and original Slide techniques by allowing direct visualization of the pedicle entry point for accurate screw placement, yet substantially enhances thoracic pedicle screw anchorage compared with those techniques. Although anchorage was lower than that of the Conventional technique, screws placed using the Modified Slide technique still withstood loads substantially exceeding those reported to occur during daily activities.

## 1 Introduction

Thoracic pedicle screw instrumentation is widely used for the treatment of spinal trauma, tumors, infections, and deformities. However, thoracic pedicle screw placement remains technically challenging and carries a significant risk of screw misplacement [1, 2, 3]. Compared to the lumbar spine, thoracic pedicles are smaller and show greater variability in shape and size [4, 5, 6]. In addition, the margin for error is low due to the close proximity of the spinal cord and nerve roots to the pedicle walls [7, 8]. Thus, thoracic pedicle screw placement demands high precision to minimize the risk of injury to adjacent neurological, vascular, and visceral structures. Several free‐hand, fluoroscopy‐assisted, navigated, and robot‐assisted techniques have been described, but none has achieved universal acceptance [2, 9].

Apart from navigated and robot‐assisted screw placement, three main techniques for thoracic pedicle screw insertion have been described in the literature [10, 11]. A first, widely used approach—referred to as the Conventional technique in this study—involves identifying the screw entry point based on anatomical landmarks or fluoroscopic guidance, creating a small cortical pilot hole using a burr or starter awl, and subsequently probing the pedicle [12]. Alternatively, some surgeons prefer drilling the entire screw path rather than using a probe. Second, the Funnel technique, first described by Gaines in 2000 [13], has been widely adopted to reduce the risk of screw misplacement. This technique involves removal of a 10‐mm section of the posterior cortex of the lamina overlying the posterior projection of the pedicle, using a burr or Leksell rongeur. The underlying cancellous bone is then removed with a small spoon curette until the cortical walls of the pedicle can be palpated and visualized. These cortical walls subsequently serve as a funnel, enabling safe insertion of the pedicle probe through the pedicle isthmus. Third, in 2014, Vialle et al. [14] introduced the Slide technique to facilitate safe thoracic pedicle screw placement, particularly in severe spinal deformities. In this technique, the posterior cortex of the transverse process is decorticated, and the underlying cancellous bone is removed; the exposed anterior cortex of the transverse process is then used as a “slide” and followed medially to safely identify the pedicle entry point. The authors of the present study have modified the original Slide technique (see *Thoracic pedicle screw insertion techniques*) to minimize decortication of the posterior cortex of the transverse process, as extensive bone removal at the screw entry point may compromise screw anchorage.

An ideal pedicle screw insertion technique should not only ensure safe and accurate screw placement but also provide sufficient screw anchorage to prevent loosening and construct failure. However, the impact of different screw insertion techniques on thoracic pedicle screw anchorage has not yet been systematically evaluated. In this biomechanical study, we compared screw anchorage following pedicle screw insertion using the Modified Slide, Slide, Funnel, and Conventional techniques in human cadaveric vertebrae under cyclic loading. We hypothesized that screws inserted using the Modified Slide technique would withstand a higher number of load cycles than those placed with the Slide and Funnel techniques, and a comparable number of cycles compared to those placed with the Conventional technique.

## 2 Materials and Methods

### 2.1 Specimens

Forty‐five fresh‐frozen human thoracic vertebrae (T4–T11) obtained from 12 different donors (6 males, 6 females; median age 68 years (IQR 16); range, 37–90 years) were used in this study. All donors had provided written informed consent for the use of their bodies for scientific and educational purposes prior to death. In accordance with national and institutional regulations, no additional formal ethical approval was required for this cadaveric study. Quantitative computed tomography (qCT) was performed using a GE LightSpeed VCT 16‐slice scanner (GE Healthcare, Waukesha, WI, USA) with a calibration phantom (European Forearm Phantom, QRM GmbH, Moehrendorf, Germany) to assess trabecular bone mineral density (BMD) of each vertebra and to rule out structural abnormalities such as fractures, infections, or neoplastic disease. Median BMD was 101.8 mg/cm^3^ (IQR 42.1). The double shrink‐wrapped specimens were stored at −20°C and thawed for 12 h at 6°C prior to preparation. All soft tissues, including the intervertebral discs, were removed and single vertebral bodies were isolated.

### 2.2 Experimental Design

**FIGURE 1:**
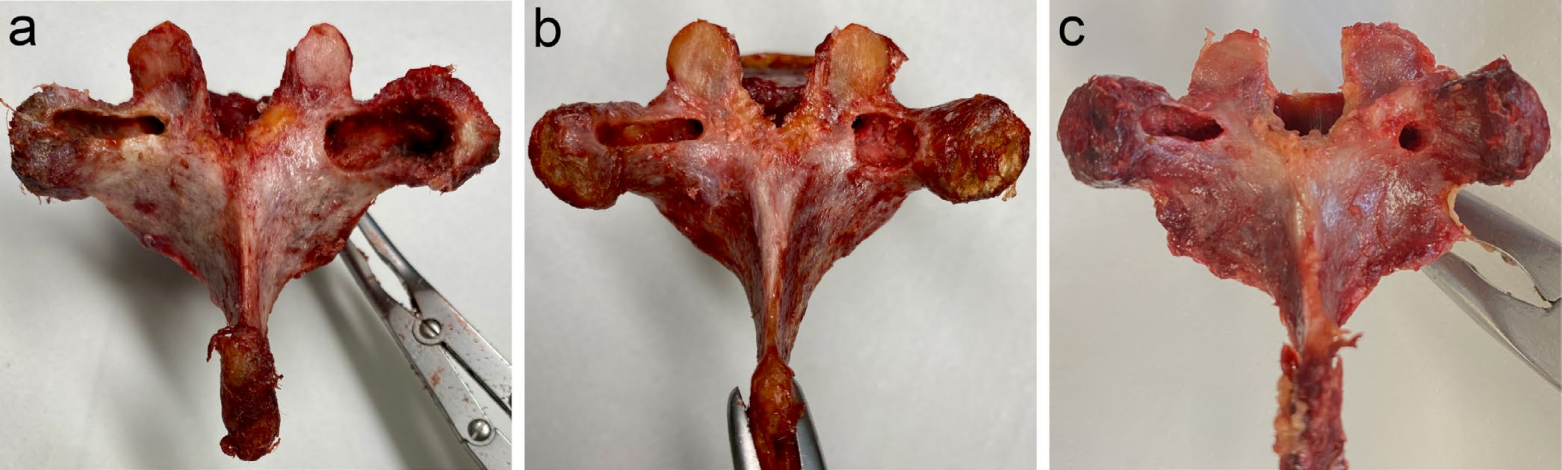
Representative specimens from experimental groups for paired comparisons: (a) Modified Slide (left side) versus Slide (right side); (b) Modified Slide (left side) versus Funnel (right side); and (c) Modified Slide (left side) versus Conventional (right side) techniques.

In this study, screw anchorage of thoracic pedicle screws placed using the Slide technique as modified by the authors (Modified Slide technique) was compared to that achieved with three established techniques described in the literature: the Slide, Funnel, and Conventional techniques. Three experimental groups were formed, each consisting of 15 thoracic vertebrae. For paired comparisons within each vertebra, one pedicle was randomly assigned to receive a screw placed using the Modified Slide technique, while the contralateral pedicle received an identical screw placed using the respective established technique (Group 1: Slide; Group 2: Funnel; Group 3: Conventional) (Figure 1). This experimental design enabled standardized, paired comparisons of the screw placement techniques while controlling for potential confounding factors such as specimen variability, particularly in bone quality and pedicle morphology.

### 2.3 Thoracic Pedicle Screw Insertion Techniques

All pedicle screws were placed by the same experienced spine surgeon (R.A.L.), who was familiar with all four screw placement techniques and had applied them in clinical practice on a regular basis. Monoaxial, cannulated pedicle screws (Verticale, Silony Medical International AG, Frauenfeld, Switzerland) of various dimensions (Ø 4.5 × 35 mm, 4.5 × 40 mm, 5.2 × 35 mm, 5.2 × 40 mm, 6.2 × 40 mm, 6.2 × 45 mm) were used to accommodate different pedicle sizes. Screw dimensions were determined by the surgeon based on multiplanar CT reconstructions of the pedicles, with identical screw sizes selected for the left and right pedicles to eliminate screw size as a confounding factor in paired comparisons within each vertebra. Mediolateral (5.6 mm (IQR 1.9) vs. 5.8 mm (IQR 1.7), *p* = 0.294) and craniocaudal (13.5 mm (IQR 3.3) vs. 13.5 mm (IQR 3.0), *p* = 0.483) pedicle diameters, as measured on multiplanar CT reconstructions, did not differ significantly between the pedicles instrumented using the Modified Slide technique and those instrumented using the established techniques across all specimens. Similarly, no significant differences in pedicle diameters were observed within the individual experimental groups.

The specific techniques for thoracic pedicle screw placement were as follows.

#### 2.3.1 Slide Technique

Screw placement using the original Slide technique was performed according to the description by Vialle et al. [14]. Briefly, the posterior cortex of the transverse process was first decorticated using a 4‐mm high‐speed burr (Fine Diamond, Stryker Corporation, Kalamazoo, MI, USA). The underlying cancellous bone was then removed with a 3‐mm spoon curette to expose the anterior cortex of the transverse process. The exposed anterior cortex was subsequently used as a ‘slide’ and followed medially to safely identify the pedicle entry point. Finally, the pedicle was cannulated using a standard pedicle probe.

#### 2.3.2 Modified Slide Technique

**FIGURE 2:**
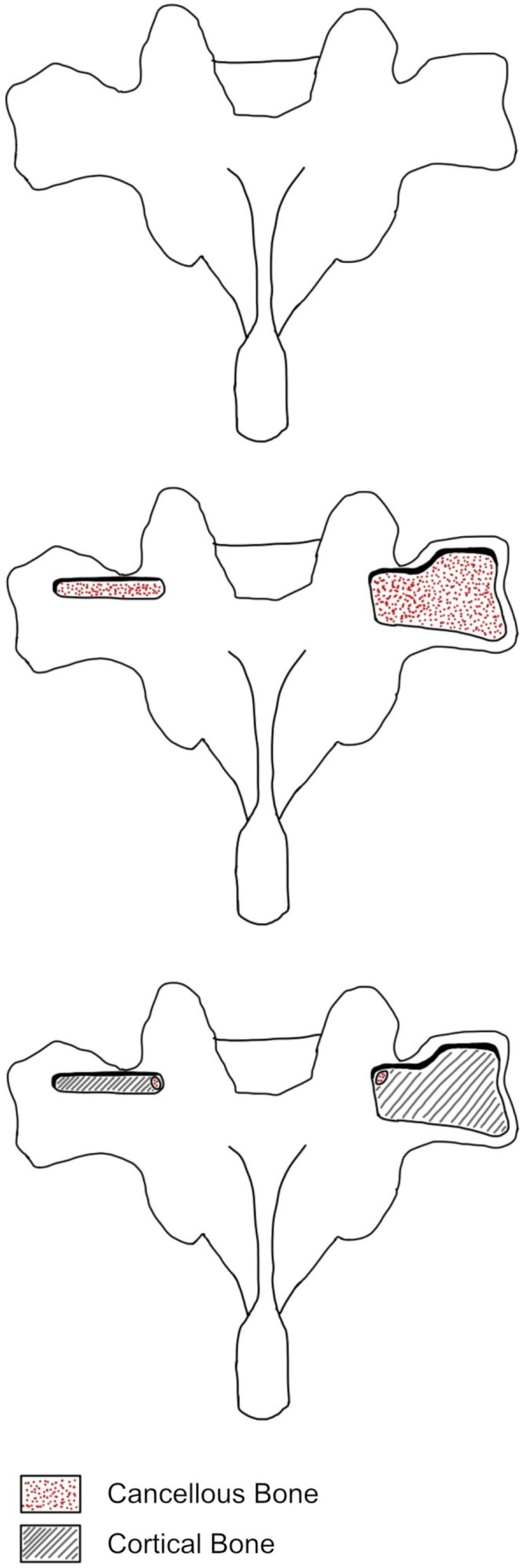
Schematic illustration of the Modified Slide technique (left side) compared to the original Slide technique (right side). The Modified Slide technique involves less removal of cortical bone at the screw entry point. See *Thoracic pedicle screw insertion techniques* for procedural details.

Since extensive cortical bone removal at the screw entry point may compromise screw anchorage, the original Slide technique was modified by the authors of the present study to minimize decortication of the posterior cortex while preserving the advantages of the original method. In the modified technique, rather than removing nearly the entire posterior cortex, a narrow horizontal burr path was created. Specifically, a cortical breach was made at the presumed screw entry point (as defined for the Conventional technique) using a 4‐mm high‐speed burr, and decortication was then extended laterally to create a narrow horizontal path (Figures 1 and 2). A 4‐mm burr was chosen to match the craniocaudal diameter of the horizontal decortication path to the diameter of the pilot hole created by the standard starter awl in the Conventional technique. The craniocaudal extent of the decortication was thus defined by the burr diameter. The mediolateral extent, similar to the original Slide technique, is not exactly predefined in millimeters but, in the Modified Slide technique, typically extends approximately 15–20 mm laterally from the entry point. This extent is generally somewhat smaller than in the original Slide technique, as schematically illustrated in Figure 2. The subsequent steps were essentially the same as those described in the original technique: The underlying cancellous bone was removed using a small spoon curette or by gently sweeping the non‐rotating burr from medial to lateral to expose the anterior cortex of the transverse process. This exposed cortex was then used as a “slide” and followed medially to safely identify the pedicle entry point. To prevent perforation of the anterior cortex, the convex, blunt side of the spoon curette was oriented toward the anterior cortex during medial sliding. Upon reaching cancellous bone at the pedicle entry, the curette was rotated 180° laterally to avoid medial perforation, and subsequently gently rotated side‐to‐side to confirm correct positioning by tactile feedback. Despite the reduced decortication, the pedicle entry point could still be directly visualized without difficulty. Finally, pedicle cannulation was performed starting at the identified pedicle entry point.

#### 2.3.3 Funnel Technique

Screw placement using the Funnel technique was performed according to the original description by Gaines [13]. Briefly, a 10‐mm section of the posterior cortex of the lamina overlying the posterior projection of the pedicle was removed using a Leksell rongeur and a burr. The underlying cancellous bone was then removed with a 3‐mm spoon curette until the cortical walls of the pedicle could be palpated and visualized. These cortical walls subsequently served as a funnel, enabling safe pedicle cannulation.

#### 2.3.4 Conventional Technique

For screw placement using the Conventional technique, a 4‐mm cortical pilot hole was created at the screw entry point using a standard starter awl (Verticale Triangular Awl, Silony Medical International AG, Frauenfeld, Switzerland), followed by pedicle cannulation. The screw entry point was determined approximately 2–3 mm lateral to the midline of the superior articular facet (medial‐lateral direction) and 3 mm caudal to the junction of the lateral margin of the superior articular facet and the transverse process (cranial‐caudal direction) [15, 16].

For all screw placement techniques, the pedicle was cannulated using a standard straight pedicle probe (Verticale Awl, Silony Medical International AG, Frauenfeld, Switzerland). Screw placement was performed in isolated vertebrae with all soft tissues removed, allowing direct visualization. After cannulation, pedicle integrity was systematically assessed by direct visualization and probing with a ball‐tipped feeler. In addition, a guide wire was inserted through the prepared path to verify a correct intrapedicular and straightforward trajectory under lateral and axial fluoroscopic views. Cannulated pedicle screws were then inserted over the guide wire to prevent deviation from the prepared path. After screw placement, pedicle wall integrity was again assessed by direct inspection of all pedicle walls, and additional lateral and axial fluoroscopic images were obtained to confirm correct screw placement. No pedicle breaches occurred in any specimen.

### 2.4 Cyclic Loading

**FIGURE 3:**
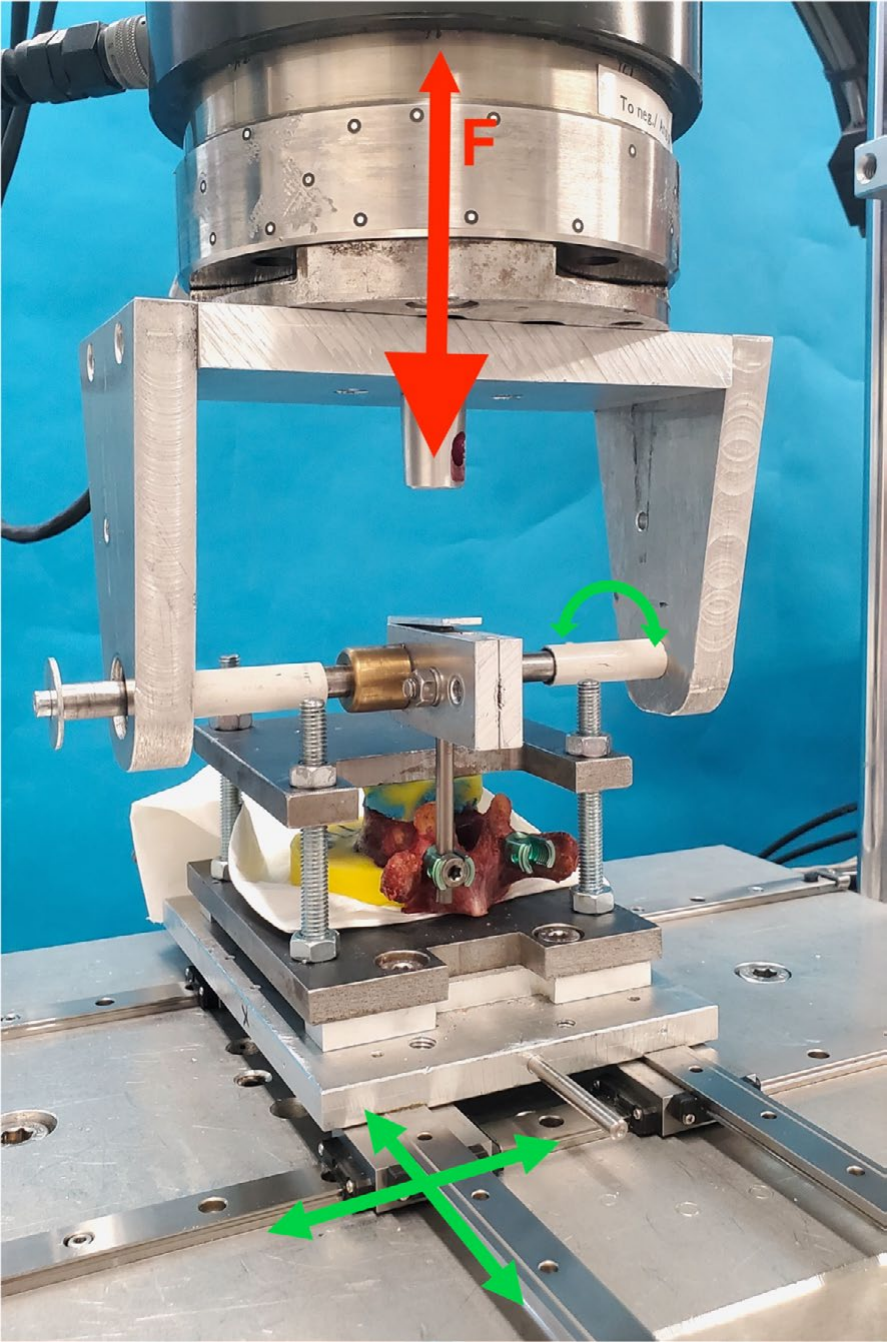
Biomechanical test setup for cyclic loading. Craniocaudal loading (F, red arrow) was applied via the actuator of the material testing apparatus. Pedicle screws were connected to the actuator with a lever arm to induce axial loading with a bending moment (green curved arrow). Specimens were mounted on an *x*–*y* table allowing translation in the anterior–posterior and medial–lateral directions (green straight arrows).

For biomechanical testing, the superior and inferior endplates of each vertebra were potted in polymethylmethacrylate (PMMA; Technovit 3040, Heraeus Kulzer GmbH, Wehrheim, Germany) to facilitate specimen fixation in the material testing apparatus. The potted vertebrae were mounted onto an *x*–*y* plane bearing table, allowing translation in the anterior–posterior and medial–lateral planes during cyclic loading (Figure 3). The pedicle screws were connected to the actuator of the servohydraulic material testing apparatus via a straight titanium rod (Ø 5.5 mm, Verticale, Silony Medical International AG, Frauenfeld, Switzerland). The rod was attached to the screws with a lever arm of 15 mm relative to the rotational axis to approximate the physiological rotational axis of an instrumented spine and to combine axial loading with an induced bending moment [17, 18].

Each pedicle screw was subjected to cyclic craniocaudal loading using a servohydraulic biaxial material testing machine (858 Mini Bionix II, MTS, Eden Prairie, MN, USA), with stepwise increasing loads until either pedicle screw loosening occurred or a maximum load level of 750 N was reached. The order of testing (screw in the left or right pedicle first) was determined by random assignment for each vertebra. Testing was performed in displacement control mode (5 mm/s) with force limits applied. The initial load ranged from −50 N (tension) to +50 N (compression), with the compressive load increased by 5 N every 100 cycles. Load and displacement were continuously recorded throughout testing, and the slope of the force curve was continuously calculated and monitored in real time using an automated algorithm. The onset of screw loosening was defined as a plateau in force within the low loading range (−10 N to +10 N), corresponding to no change in slope over a predefined time window (0.04 s, corresponding to an actuator displacement of 0.2 mm), based on pilot experiments.

### 2.5 Statistical Analysis

All statistical analyses were performed using IBM SPSS Statistics, version 29 (IBM Corp., Armonk, NY, USA). Normality was assessed using the Shapiro–Wilk test. As several variables were not normally distributed, descriptive statistics are presented as median (interquartile range [IQR]), and paired comparisons between screw insertion techniques were performed using the Wilcoxon signed‐rank test. As the paired differences in load cycles and corresponding load levels were normally distributed, paired *t*‐tests were additionally performed as a sensitivity analysis for these outcomes and yielded comparable results. A formal a priori sample size calculation was not performed due to the absence of directly comparable data for the investigated screw insertion techniques under cyclic craniocaudal loading. A post hoc power analysis for the paired comparisons of load cycles until loosening was therefore performed based on the observed group means, standard deviations, and within‐pair correlation coefficients, yielding powers of > 99% for the Modified Slide versus Slide and Modified Slide versus Funnel comparisons, and 88% for the Modified Slide vs. Conventional comparison. Comparisons across the three experimental groups were performed using the Kruskal–Wallis test for continuous variables, with post hoc Mann–Whitney *U* tests and Bonferroni correction when significant; for categorical variables, the Pearson Chi‐square test was used. To assess factors associated with the number of load cycles until loosening following screw insertion using the Modified Slide technique, all screws placed with this technique across the three comparison groups were pooled (*n* = 45). Univariate linear regression analyses were conducted using a generalized estimating equation (GEE) model to account for clustering within donors. A *p*‐value < 0.05 was considered statistically significant.

## 3 Results

A total of 90 pedicle screws were tested in 45 human thoracic vertebrae (T4–T11), resulting in 30 screws and 15 vertebrae per experimental group. Specimen characteristics and biomechanical outcomes are summarized in Table 1. No significant differences were observed across the three experimental groups for age, sex, or Modified Slide outcomes. BMD differed significantly across groups (Kruskal–Wallis test, *p* = 0.025). Post hoc pairwise comparisons with Bonferroni correction showed a significant difference between the MS versus Slide and MS versus Conventional groups (*p* = 0.021), whereas no significant differences were observed between the MS versus Slide and MS versus Funnel groups (*p* = 0.330) or the MS versus Funnel and MS versus Conventional groups (*p* = 0.816).

**TABLE 1:** Specimen characteristics and biomechanical test results.

Group	Age (years)	Sex (M/F), *n*	BMD (mg/cm^3^)	Modified Slide technique (MS)		Contralateral control techniques		*p*	
				Cycles to loosening	Load at loosening (*N*)	Cycles to loosening	Load at loosening (*N*)	*p* (cycles)	*p* (load)
All (*n* = 45)	68 (16)	20/25	101.8 (42.1)	7642 (4346)	430 (215)	5484 (5590)	320 (263)		
MS versus Slide (*n* = 15)	65 (51)	8/7	92.6 (29.2)	7239 (4432)	410 (220)	3854 (2609)	240 (130)	**< 0.001**	**< 0.001**
MS versus Funnel (*n* = 15)	80 (19)	7/8	103.5 (47.4)	6448 (6260)	370 (315)	4277 (3349)	260 (165)	**< 0.001**	**< 0.001**
MS versus Conventional (*n* = 15)	68 (8)	5/10	120.0 (38.6)	8064 (2834)	445 (145)	9123 (3936)	500 (195)	**0.008**	**0.008**
*p* across groups	0.539	0.533	**0.025**	0.754	0.784				

*Note:* Data are presented as median (interquartile range) for continuous variables and as counts for categorical variables. Paired comparisons between Modified Slide and contralateral control screws (Slide, Funnel, and Conventional techniques) were performed using the Wilcoxon signed‐rank test. Comparisons across the three experimental groups were performed using the Kruskal–Wallis test for continuous variables (age, BMD, and Modified Slide outcomes) and the Pearson Chi‐square test for categorical variables (sex). Bold values indicate statistically significant differences (*p* < 0.05).

Abbreviations: BMD, bone mineral density; F, female; M, male; MS, Modified Slide technique; Slide, original Slide technique; Funnel, Funnel technique; Conventional, Conventional technique.

### 3.1 Modified Slide Versus Slide Technique

**FIGURE 4:**
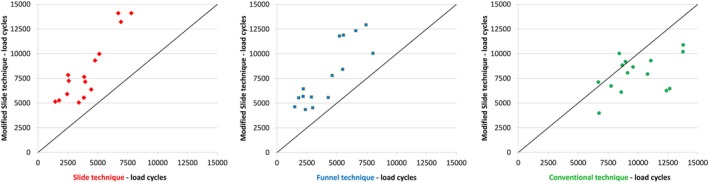
Scatterplots of paired comparisons of load cycles until loosening: Left, Modified Slide versus Slide techniques; middle, Modified Slide versus Funnel techniques; right, Modified Slide versus Conventional techniques. Each data point represents a paired comparison of two pedicle screws placed within the same vertebra using different techniques. Data points above the diagonal reference line indicate a higher number of load cycles until loosening for the Modified Slide technique compared with the respective alternative.

**FIGURE 5:**
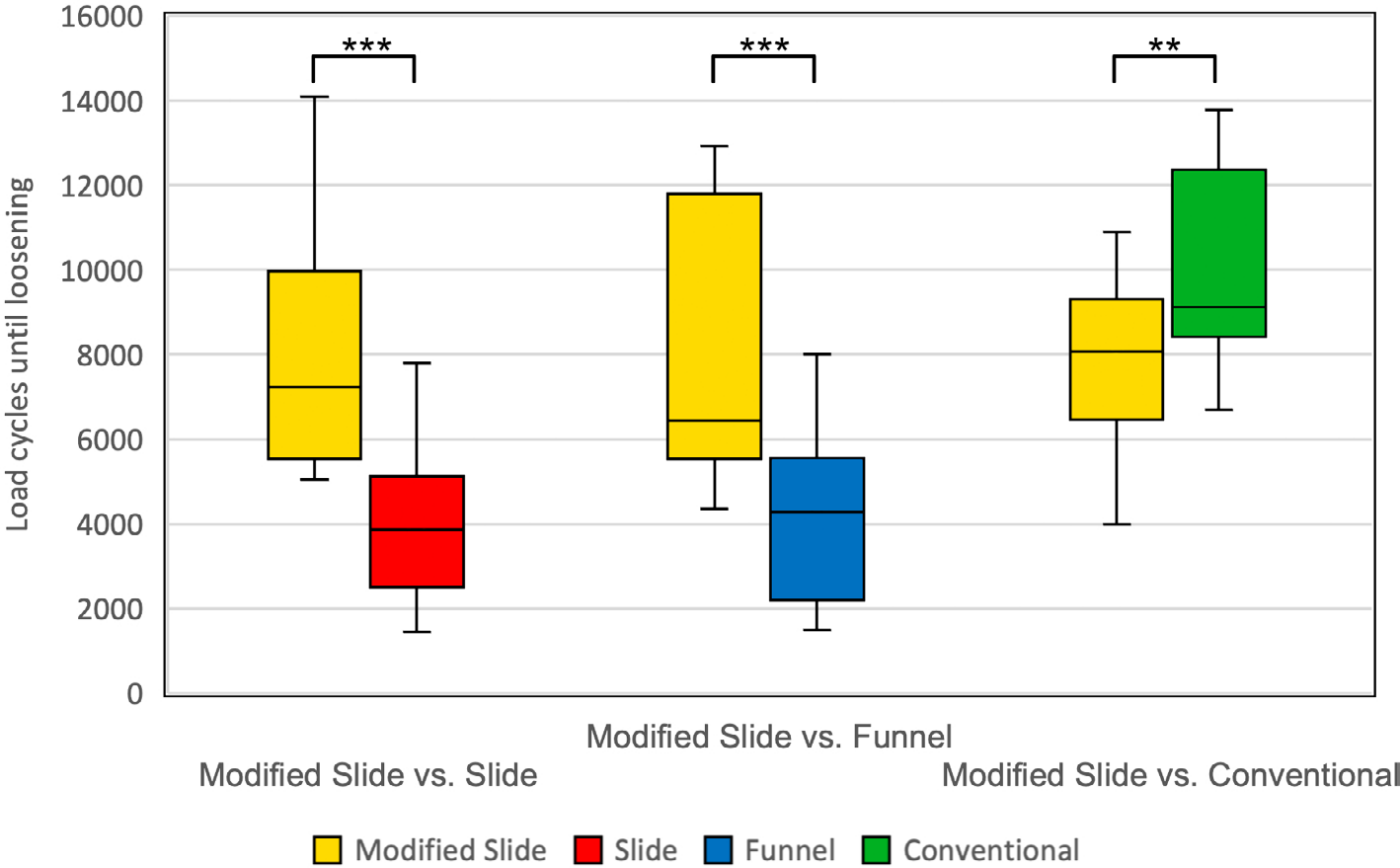
Boxplots of paired comparisons of load cycles until loosening: Left, Modified Slide versus Slide techniques; middle, Modified Slide versus Funnel techniques; right, Modified Slide versus Conventional techniques. Boxes represent the interquartile range (IQR), horizontal lines indicate medians, and whiskers show minimum and maximum values. Asterisks indicate statistically significant differences (**p < 0.01; ***p < 0.001).

In all 15 vertebrae tested, screws placed with the Modified Slide technique performed better than contralateral control screws placed with the Slide technique in terms of the number of load cycles until loosening (Figure 4). Specifically, Modified Slide screws withstood a significantly higher number of load cycles until loosening than contralateral Slide screws (7239 (4432) vs. 3854 (2609) load cycles, *p* < 0.001; Figure 5), corresponding to load levels at loosening of 410 N (220) and 240 N (130), respectively (*p* < 0.001). Notably, two screws implanted using the Modified Slide technique did not loosen until reaching the maximum load of 750 N.

### 3.2 Modified Slide Versus Funnel Technique

As observed in the previous comparison, Modified Slide screws performed better than contralateral Funnel screws across all 15 vertebrae tested (Figure 4). Specifically, Modified Slide screws withstood a significantly higher number of load cycles until loosening than contralateral Funnel screws (6448 (6260) vs. 4277 (3349) load cycles, *p* < 0.001; Figure 5), corresponding to load levels at loosening of 370 N (315) and 260 N (165), respectively (*p* < 0.001).

### 3.3 Modified Slide Versus Conventional Technique

In only four out of 15 vertebrae, the Modified Slide screws demonstrated a greater number of load cycles until loosening, whereas in 11 vertebrae, screws placed with the Conventional technique performed better (Figure 4). Modified Slide screws withstood a significantly lower number of load cycles until loosening than conventionally implanted screws (8064 (2834) vs. 9123 (3936) load cycles, *p* = 0.008; Figure 5), corresponding to load levels at loosening of 445 N (145) and 500 N (195), respectively (*p* = 0.008).

### 3.4 Factors Associated With Screw Anchorage of Screws Placed Using the Modified Slide Technique

All screws placed using the Modified Slide technique across the three comparison groups (*n* = 45) were pooled to assess factors associated with screw anchorage. Univariate analysis revealed that only BMD was significantly associated with the number of load cycles until loosening (*p* < 0.001). All other variables, including age (*p* = 0.286), sex (*p* = 0.095), screw diameter (*p* = 0.625), screw length (*p* = 0.556), and medio‐lateral pedicle diameter (*p* = 0.751), showed no significant associations.

## 4 Discussion

This biomechanical study evaluated the effect of different pedicle screw insertion techniques on thoracic pedicle screw anchorage under cyclic loading conditions. Screws placed using the Modified Slide technique withstood 1.9‐fold and 1.5‐fold more load cycles until loosening than those placed with the Slide and Funnel techniques, respectively, but only 0.9‐fold the load cycles of screws inserted with the Conventional technique. Of the factors analyzed, only BMD was significantly associated with the load cycles until loosening of screws implanted with the Modified Slide technique.

From a clinical perspective, an ideal thoracic pedicle screw placement technique should ensure both safe screw insertion to minimize the risk of misplacement and sufficient screw anchorage to prevent loosening and construct failure. The Conventional technique, which involves creating a small cortical pilot hole at the screw entry point with a burr or starter awl prior to pedicle cannulation, has been widely used; however, achieving proper screw placement can be challenging in difficult anatomical situations. This is reflected by the relatively high rates of pedicle breaches reported in the thoracic spine [2, 19, 20, 21, 22].

The Funnel and Slide techniques were developed to improve the safety and accuracy of thoracic pedicle screw placement, particularly in complex anatomical scenarios such as small pedicle dimensions or severe spinal deformities. In a retrospective clinical study of 115 patients (aged 9–82 years) treated with the Funnel technique, one patient experienced transient anterior thigh numbness, and no other new neurologic deficits, vascular or pulmonary complications were observed postoperatively [23]. A total of 348 screws (diameter 4.0–7.75 mm) were placed between T5 and T12 in patients treated for fractures (*n* = 63), scoliosis (*n* = 41), and other spinal conditions. Notably, 50%–60% of screws were placed by 25 different residents during their first or second spine surgery rotation, under the supervision of five fellows and the attending surgeon who originally described the technique. A cadaveric study reported pedicle perforation rates ranging from 12.5% (junior spine surgeon without prior thoracic screw experience) to 1.4% (senior spine surgeon), with only one of 214 screws (0.5%) contacting an adjacent nerve root [24]. Similarly, in a study of Asian cadavers, an overall pedicle breach rate of 10.4% was reported for 240 thoracic pedicle screws, though only one perforation (0.4%) was deemed significant [25]. In another retrospective study, Kilicaslan et al. [26] observed significantly fewer malpositioned screws with a modified Funnel technique compared to the Conventional technique (4.6% vs. 12.0%, *p* = 0.001) in 343 spinal deformity patients with a total of 6141 screws. However, malposition was assessed using plain radiographs rather than CT imaging. This modified technique essentially represents a combination of the Funnel and Slide techniques, involving removal of the posterior cortex overlying the pedicle's posterior projection, followed by decortication of the posterior cortex of the transverse process. Regarding the Slide technique, Vialle et al. [14] used this method to place more than 18 000 thoracic pedicle screws, primarily in patients with severe spinal deformities. No patient required screw revision, and no neurologic deficits occurred related to screw malpositioning.

However, while the Funnel and Slide techniques facilitate safe screw placement, they involve more extensive decortication at the screw entry point compared to the Conventional technique, which may compromise screw anchorage. Although several studies have investigated the influence of pedicle pilot hole diameter relative to the inner and outer diameters of pedicle screws [27, 28, 29], only one study, to our knowledge, has specifically examined the impact of cortical pilot hole size at the screw entry point. In a biomechanical study using both synthetic bone and calf vertebrae, Daftari et al. [30] demonstrated that pull‐out force was more dependent on the diameter of the cortical entrance hole than on that of the pedicle pilot hole, underscoring the critical role of the posterior cortex in pedicle screw fixation. Our findings corroborate this observation: screw insertion techniques involving more extensive decortication—such as the Funnel and Slide techniques—showed substantially lower screw anchorage than the Modified Slide technique in paired comparisons, while the Modified Slide technique showed lower anchorage than the Conventional technique. Notably, the extent of posterior cortical bone removal was the key distinguishing factor between the evaluated techniques, as pedicle cannulation beyond the entry point was performed identically in all groups.

In contrast to the well‐studied topic of screw placement accuracy, the impact of different screw insertion techniques on thoracic pedicle screw anchorage has not been systematically evaluated. While previous studies have examined factors such as screw trajectory, tapping, screw diameter‐to‐pedicle width ratio, axial screw misplacement, and screw reinsertion [28, 31, 32, 33, 34, 35, 36, 37, 38], only one study has specifically evaluated the influence of the screw insertion technique itself. Using a T8 anterior column injury model and T6–T10 human spinal specimens, Huang et al. [39] performed bisegmental instrumentation with 4.5 mm × 50 mm pedicle screws placed at T7 and T9 using either the Funnel or Conventional technique (*n* = 7 specimens per group). The stiffness of the four‐screw constructs did not differ significantly between techniques. However, subsequent pull‐out testing revealed an 11.1% lower maximum pull‐out force for screws placed using the Funnel technique compared to the Conventional technique (789.09 ± 27.33 N vs. 887.93 ± 19.14 N, *p* < 0.001). In our study, we observed a substantially greater difference: the number of cycles until loosening was 34% lower for screws placed using the Funnel technique compared to the Modified Slide technique. This discrepancy might relate to the higher donor age of our specimens but may particularly be attributed to differences in biomechanical testing protocols. While Huang et al. used simple pull‐out testing without ensuring alignment between the screw axis and the pull‐out vector, our study employed cyclic loading until loosening—a method widely regarded as more appropriate for assessing screw anchorage and simulating in vivo loosening behavior, with greater sensitivity to the effects of decortication at the screw entry point.

The authors of this study modified the original Slide technique to minimize decortication at the screw entry point, aiming to improve screw anchorage while preserving the advantages of the original technique—namely, the use of the anterior cortex as a guide and direct visualization of the pedicle entry to enhance placement safety. In the modified Slide technique, decortication was limited to a narrow, horizontal path starting at the entry point and extending laterally, with the craniocaudal extent matching that of the cortical pilot hole in the Conventional technique (Figures 1 and 2). This approach specifically addresses the main challenge in thoracic pedicle screw placement—achieving mediolateral accuracy and preventing medial breaches—while accounting for the predominantly craniocaudal physiological loading pattern in vivo. In our clinical practice, the Modified Slide technique is fast, safe, and allows consistent direct visualization of the pedicle entry point despite the reduced extent of decortication. However, screws placed using the Modified Slide technique withstood approximately 12% fewer load cycles than those placed using the Conventional technique. One possible explanation is that, although the craniocaudal extent of the decortication path was identical to that of the cortical pilot hole in the Conventional technique, the additionally removed bone lateral to the screw cannot contribute effectively to load transfer during craniocaudal loading, potentially compromising anchorage. Nevertheless, screws placed with the Modified Slide technique still withstood median loads of 370–445 N until loosening, which is well above the peak loads of 200–250 N measured in patients with a telemeterized internal fixator during daily activities [40, 41] and within the upper range of the 300–450 N reported for cement‐augmented lumbar pedicle screws evaluated under identical test conditions [17, 18, 42]. However, the telemetric reference values are derived predominantly from the lumbar spine, with only a few lower thoracic levels included (T11 and T12), and direct extrapolation to the thoracic spine is therefore limited; lower loads may be expected in the thoracic spine.

This study has several notable strengths. First, it is the first biomechanical investigation to specifically evaluate the impact of several commonly used thoracic pedicle screw placement techniques—along with a novel modification—on screw anchorage. Second, the use of cyclic craniocaudal loading provided a validated and physiologically relevant testing protocol, widely regarded as the most appropriate method for assessing screw anchorage and simulating in vivo loosening [43, 44]. Third, the paired experimental design enabled direct intra‐specimen comparisons, effectively controlling for potential confounding factors such as BMD and pedicle morphology. Fourth, all screws were placed in a highly standardized manner, strictly following the original description of each technique to ensure procedural consistency across groups.

Nevertheless, this study is subject to the inherent limitations of cadaveric biomechanical testing. Although the cyclic loading protocol closely approximates physiological conditions, it cannot fully replicate the complexity of in vivo spinal loading or account for biological processes such as osseointegration. Moreover, as specimens were predominantly derived from elderly donors and the median BMD was in the osteopenic range, generalizability to patients with higher bone quality may be limited. All screw placements were performed by a single experienced surgeon, and technique‐specific proficiency differences cannot be excluded, which may limit generalizability. Furthermore, this biomechanical study was designed to isolate the effect of the insertion technique and associated extent of cortical bone removal at the screw entry point on thoracic pedicle screw anchorage. Accordingly, all screws were placed with a correct intrapedicular trajectory without pedicle wall violation. Therefore, potential accuracy‐related advantages of techniques allowing direct visualization of the pedicle entry point and their impact on placement safety and screw anchorage were not evaluated, although screw malposition may also reduce screw anchorage [37, 45, 46]. Moreover, as the Modified Slide technique minimizes posterior cortical bone removal, the reduced extent of decortication may limit the visualization window for the pedicle entry point compared to the original Slide technique, representing a potential trade‐off between improved screw anchorage and intraoperative visualization. Finally, this study did not include direct paired comparisons among the Funnel, Slide, and Conventional techniques, which would have required substantially more specimens. Instead, the Modified Slide technique was used as a reference for controlled paired intra‐vertebral comparisons with each respective control technique, as it was considered an appropriate reference combining facilitated screw placement with reduced posterior cortical bone removal. As a result, direct statistical comparisons between the Funnel, Slide, and Conventional techniques are not possible within the study design, and any indirect comparisons between these techniques should be interpreted as hypothesis‐generating. In addition, BMD differed across groups, which may further limit the interpretation of indirect comparisons between the Funnel, Slide, and Conventional techniques.

## 5 Conclusions

This study evaluated the effect of different pedicle screw insertion techniques on thoracic pedicle screw anchorage under cyclic loading. Screws placed using the Modified Slide technique withstood 1.9‐fold and 1.5‐fold more load cycles until loosening than those placed with the Slide and Funnel techniques, respectively, but only 0.9‐fold the load cycles of screws inserted with the Conventional technique. The Modified Slide technique preserves the advantage of the Funnel and original Slide techniques by allowing direct visualization of the pedicle entry point for accurate screw placement, yet substantially enhances thoracic pedicle screw anchorage compared with those techniques. Although anchorage was lower than that of the Conventional technique, screws placed using the Modified Slide technique still withstood loads substantially exceeding the peak loads reported to occur during daily activities and falling within the upper range of those reported for cement‐augmented lumbar pedicle screws tested under identical conditions. These findings suggest that the Modified Slide technique may represent a reasonable compromise between improved visualization of the pedicle entry point to facilitate correct screw placement and preservation of thoracic pedicle screw anchorage when enhanced visualization is clinically desirable, particularly in challenging anatomical situations such as small pedicles and spinal deformity.

## Author Contributions

**Julian Benko:** investigation, writing – review and editing, visualization. **Fabian Krumm:** investigation, formal analysis, writing – review and editing. **Andreas E. Ellmerer:** writing – review and editing, investigation, project administration. **Richard A. Lindtner:** writing – review and editing, investigation, conceptualization, project administration, methodology. **Anna Spicher:** writing – review and editing, investigation, conceptualization, project administration, methodology. **Romed Hoermann:** writing – review and editing, resources, project administration. **Johannes Dominikus Pallua:** writing – review and editing, formal analysis, project administration. **Werner Schmoelz:** methodology, validation, formal analysis, supervision, resources, writing – review and editing, conceptualization, investigation.

## Funding

This work was supported by a research grant provided by AO Trauma Austria.

## Conflicts of Interest

The authors declare no conflicts of interest.

## Data Availability

The data that support the findings of this study are available from the corresponding author upon reasonable request.
